# Maternal dietary resistant starch does not improve piglet’s gut and liver metabolism when challenged with a high fat diet

**DOI:** 10.1186/s12864-020-06854-x

**Published:** 2020-06-26

**Authors:** Martine Schroyen, Julie Leblois, Julie Uerlings, Bing Li, Ester Arévalo Sureda, Sébastien Massart, José Wavreille, Jérôme Bindelle, Nadia Everaert

**Affiliations:** 1 Precision Livestock and Nutrition Laboratory, Teaching and Research Centre (TERRA), Gembloux AgroBioTech, University of Liège, Passage des Déportes 2, 5030 Gembloux, Belgium; 2 Association Wallonne de l’Elevage asbl (AWÉ), Champs Elysées 4, 5590 Ciney, Belgium; 3 Laboratory of Urban and Integrated PhytoPathology, Teaching and Research Centre (TERRA), Gembloux AgroBioTech, University of Liège, 5030 Gembloux, Belgium; 4 Production and Sectors Department, Walloon Agricultural Research Centre (CRA-W), 5030 Gembloux, Belgium

**Keywords:** Metabolic programming, Microbiota, Maternal programming

## Abstract

**Background:**

In the past several years, the use of resistant starch (RS) as prebiotic has extensively been studied in pigs, and this mostly in the critical period around weaning. RS is believed to exert beneficial effects on the gastrointestinal tract mainly due to higher levels of short chain fatty acids (SCFAs) and an improved microbiota profile. In this study, sows were fed digestible starch (DS) or RS during late gestation and lactation and the possible maternal effect of RS on the overall health of the progeny was assessed. Since RS is also described to have a positive effect on metabolism, and to investigate a metabolic programming of the progeny, half of the piglets per maternal diet were assigned to a high fat diet from weaning on to 10 weeks after.

**Results:**

No bodyweight differences were found between the four experimental piglet groups. The high fat diet did however impact back fat thickness and meat percentage whereas maternal diet did not influence these parameters. The impact of the high fat diet was also reflected in higher levels of serum cholesterol. No major differences in microbiota could be distinguished, although higher levels of SCFA were seen in the colon of piglets born from RS fed sows, and some differences in SCFA production were observed in the caecum, mainly due to piglet diet. RNA-sequencing on liver and colon scrapings revealed minor differences between the maternal diet groups. Merely a handful of genes was differentially expressed between piglets from DS and RS sows, and network analysis showed only one significant cluster of genes in the liver due to the maternal diet that did not point to meaningful biological pathways. However, the high fat diet resulted in liver gene clusters that were significantly correlated with piglet diet, of which one is annotated for lipid metabolic processes. These clusters were not correlated with maternal diet.

**Conclusions:**

There is only a minor impact of maternal dietary RS on the progeny, reflected in SCFA changes. A high fat diet given to the progeny directly evokes metabolic changes in the liver, without any maternal programming by a RS diet.

## Background

In the past several years, diet supplementation with resistant starch (RS), which acts as prebiotic, has extensively been studied in the pig industry. Prebiotics escape enzymatic digestion due to their chemical and physical properties and are fermented in the large intestine. There, they can promote the establishment of beneficial microbiota [1]. Most of the studies applying RS in the diet of pigs at the time of weaning report an improvement of gut health and function [2]. Fermentation of RS results in an increased amount of short chain fatty acids (SCFAs). These SCFAs, of which butyrate is the most beneficial for health, are able to prevent the overgrowth of pathogenic bacteria, and thus endorse health-promoting bacteria [3]. Changes in microbial action due to RS seems to mediate, at least in part, the expression of pro-inflammatory pathways in caecum and colon [4]. RS in the diet initiates the suppression of both adaptive and innate immune response pathways in the colon [5]. Fan et al. [6] found that a diet rich in RS would increase colonic *IL10* abundance, which may protect the colon from developing inflammation.

In addition to playing a role in gut health, RS has been fairly well characterized for its glycaemic control properties, elicited by an attenuation of blood glucose and insulin sensitizing properties [7]. Genes related to lipid synthesis in the liver and adipose tissue were found to be differentially expressed when comparing pigs fed a control diet versus those fed a diet rich in RS [8]. If dietary fibre supplementation in addition to improving gut health also limits the incidence of metabolic disorders such as non-alcoholic fatty liver disease, then it can have major implications with regard to overall health [9].

Maternal nutritional programming has been described to have long term effects, including alterations in intestinal function and defence mechanisms, lasting until weaning or even until sexual maturity is reached [10, 11]. For example, short-chain fructo-oligosaccharides fed during the last third of gestation and lactation to sows improved the progeny’s ileal cytokine secretion and raised caecal goblet cell numbers as well as the responsiveness of the piglets against vaccination [11]. Feeding polyunsaturated fatty acids to sows 150 days prior to farrowing improved intestinal glucose absorption in the small intestine of the piglets [12]. Pregelatinized waxy maize starch combined with guar gum as an additive in the feed of gestating sows was found to reduce intestinal permeability and inflammation in 14-day-old suckling piglets [13]. In mice, giving an oligofructose during pregnancy and lactation of obese mice improved the metabolism in offspring so that they could counteract the negative nutritional programming associated with maternal obesity [14]. Adding the probiotic Lactobacillus casei or the prebiotic inulin to a high fructose maternal diet during pregnancy and lactation could neutralize negative effects due to the high fructose such as hypertension in the offspring [15]. So far, research on dietary RS in pigs is mainly done applying direct supplementation of the dietary fibre. However, as in aforementioned studies, it is interesting to see if RS added to the maternal diet during the gestation and lactation period can improve gut microbiota, gut health and/or metabolism of the offspring.

Previously, we investigated if RS could alter the microbiota of the sows during late gestation and lactation and if this alteration would lead to a modified microbiota profile in the progeny at the time of weaning, and subsequently improve gut health. Although RS influenced the microbiota profile of the sows during gestation, this difference disappeared at lactation and was also not reflected in the microbiome of the progeny at the time of weaning. At the time of weaning, no major differences were observed in the progeny with regard to gut morphology nor when looking at the expression of selected immune genes [16]. However, in germ-free mice, thus even in the absence of microbiota, Bindels et al. [17] have shown that RS alters several bile acids and modulate the immune function in adipose tissue. Therefore, in this study, even in the absence of changes in microbiota of the sows or progeny at time of weaning, we investigated in more detail the response of a maternal RS diet on the colon and liver transcriptome of the progeny. To examine potential metabolic programming, after weaning, a high fat diet was assigned to half of the offspring, as RS has also been shown to have direct beneficial effects on lipid metabolism [18]. Our hypothesis was that giving RS through a maternal diet, could potentially help regulating the metabolism of the progeny to such an extent that piglets cope better with a high fat challenge later in life. A high fat diet is also known to affect microbiota [19] and therefore it is possible that an effect of RS will be observed at the intestinal and/or metabolic level when piglets are challenged. Besides growth parameters, carcass traits, SCFA production and microbiota composition at 10 weeks post-weaning, the metabolic influence of RS was examined by measuring gene expression in colon and liver in piglets that received a control or high fat diet during those 10 weeks after weaning.

## Results

### Growth performances

Piglets were selected based on similar weights at time of weaning (28 days old) with an average of 6.17 ± 0.26 kg for piglets from RS sows and 5.97 ± 0.25 kg for piglets from DS sows. Neither the maternal nor the piglet diet impacted the weekly bodyweight gain of the animals from this moment till slaughtering (Supplementary Figure 1).

The HF challenge impacted both back fat thickness and meat percentage (*p* < 0.001, Table 1), as challenged piglets had a higher back fat thickness (5.9 ± 0.2 mm for CON piglets vs 7.9 ± 0.4 mm for HF piglets) and subsequent lower meat percentage (63.5 ± 0.3% for CON piglets vs 62.0 ± 0.3% for HF piglets). Muscle thickness was not impacted by the maternal or high fat treatments.

**Table 1:** Back fat thickness (mm), muscle thickness (mm), meat percentage (%) and bodyweight (kg) of piglets after the high fat challenge (*n* = 9 for DS CON, DS HF, RS CON and *n* = 11 for RS HF)

Treatment	Back fat thickness	Muscle thickness	Meat percentage	Bodyweight at 10 weeks Post-weaning
DS CON	6.0	42.3	63.5	37.6
DS HF	7.1	44.0	62.7	35.4
RS CON	5.8	40.6	63.4	38.0
RS HF	8.5	42.5	61.5	36.2
Global SEM	0.3	1.0	0.2	0.9
***P*-values**				
Maternal treatment	0.22	0.43	0.11	0.73
Piglet treatment	**< 0.001**	0.37	**< 0.001**	0.28
Interaction	0.10	0.94	0.19	0.92

*DS CON* digestible starch maternal diet combined with a control piglet diet, *DS HF* digestible starch maternal diet combined with a high fat piglet diet, *RS CON* resistant starch maternal diet combined with a control piglet diet, *RS HF* resistant starch maternal diet combined with a high fat piglet diet, *SEM* standard error to the mean

### Cholesterol and triglycerides analyses

Total cholesterol, triglycerides and HDL concentrations increased with HF challenge (Table 2), while the ratio of LDL over total cholesterol decreased. Moreover, levels of LDL were lower for piglets born from RS sows compared to DS sows, leading to a lower LDL/TC ratio for these piglets.

**Table 2:** Total cholesterol (mg/dl), triglycerides (mg/dl), high density lipoprotein (mg/dl), low density lipoprotein (mg/dl) and ratio in fasted pigs’ serum after 6 weeks of HF challenge (*n* = 7/treatment)

Treatment	TC	TG	HDL	LDL	ratio LDL/TC
DS CON	95.3^a,b^	40.7^b^	46.3^b^	40.8^a^	42.6^a^
DS HF	105.3^a^	44.0^b^	58.7^a^	37.8^a,b^	35.9^b^
RS CON	86.4^b^	38.3^b^	43.8^b^	35.0^ab^	40.4^a,b^
RS HF	101.7^a^	54.0^a^	59.6^a^	31.3^b^	30.1^c^
Global SEM	2.66	1.91	1.90	1.55	1.27
***P*-values**					
Maternal treatment	0.21	0.26	0.78	**0.05**	**0.04**
Piglet treatment	**0.01**	**< 0.01**	**< 0.001**	0.27	**< 0.001**
Interaction	0.59	0.07	0.53	0.90	0.35

^a, b^ Between treatments for every parameter measured, values having a different superscript letter are significantly different (*p* < 0.01)

*DS CON* digestible starch maternal diet combined with a control piglet diet, *DS HF* digestible starch maternal diet combined with a high fat piglet diet, *RS CON* resistant starch maternal diet combined with a control piglet diet, *RS HF* resistant starch maternal diet combined with a high fat piglet diet, *SEM* standard error to the mean. *TC* total cholesterol, *TG* triglycerides, *HDL* high density lipoprotein, *LDL* low density lipoprotein

### Short chain fatty acids (SCFAs)

Maternal treatment impacted the total production of SCFA in the caecum and proximal colon of the piglets (Table 3), piglets born from RS sows having a higher SCFA production than piglets born from DS sows. However, in the caecum, an interaction between sow and piglet treatment was observed (*p* = 0.04) as only within the HF treatment, pigs born from RS sows had a significantly (*p* < 0.01) higher total SCFA production than pigs born from DS sows. In the caecum of piglets, acetate and propionate productions were impacted by the high fat challenge, as propionate production was increased in challenged pigs at the expense of acetate. In the distal colon, acetate production was affected differently depending on sow treatment (interaction effect *p* = 0.02): within the DS pigs, challenged pigs had a significantly lower acetate production (*p* = 0.048) than CON pigs; within the HF treatment, RS derived HF piglets had a significantly higher acetate production than DS derived HF piglets (*p* = 0.03). Butyrate production was not affected by either treatment within all intestinal segments.

**Table 3:** Total SCFA production and molar ratios of acetate, propionate and butyrate in the large intestinal contents of the piglets born from DS or RS sows and fed CON or HF diets

	Maternal treatment	Piglet treatment	Total SCFA (mg/g)	% acetate	% propionate	% butyrate
Caecum	DS	CON (*n* = 8)	9.24^ab^	62.49	27.03	10.08
		HF (*n* = 9)	7.62^b^	59.18	29.5	9.73
	RS	CON (*n* = 9)	9.29^a^	59.52	28.24	10.19
		HF (*n* = 12)	10.09^a^	56	29.89	9.19
		Global SEM	0.31	0.85	0.44	0.27
	*P*-values	P sow	**0.03**	0.06	0.36	0.70
		P piglet	0.48	**0.04**	**0.02**	0.23
		P interaction	**0.04**	0.95	0.64	0.55
Proximal colon	DS	CON (*n* = 8)	8.89	60.4	28.02	10.54
		HF (*n* = 9)	8.76	58.3	28.55	10.26
	RS	CON (*n* = 9)	9.58	59.3	28.69	10.53
		HF (*n* = 12)	10.29	56.2	29.31	9.5
		Global SEM	0.23	0.76	0.46	0.27
	*P*-values	P sow	**0.01**	0.30	0.47	0.49
		P piglet	0.50	0.09	0.56	0.24
		P interaction	0.33	0.74	0.96	0.50
Distal colon	DS	CON (*n* = 8)	7.93	55.01^a^	22.64	14.23
		HF (*n* = 9)	8.89	51.03^b^	23.23	16.24
	RS	CON (*n* = 9)	9.35	52.51^ab^	21.97	16.36
		HF (*n* = 12)	8.61	54.91^a^	22.95	15.11
		Global SEM	0.22	0.68	0.36	0.44
	*P*-values	P sow	0.19	0.60	0.53	0.57
		P piglet	0.80	0.55	0.29	0.67
		P interaction	0.06	**0.02**	0.79	0.07

*DS* digestible starch diet, *RS* resistant starch diet, *CON* control diet, *HF* high fat diet, *SCFA* short chain fatty acids

### Microbiota composition

**Fig. 1:**
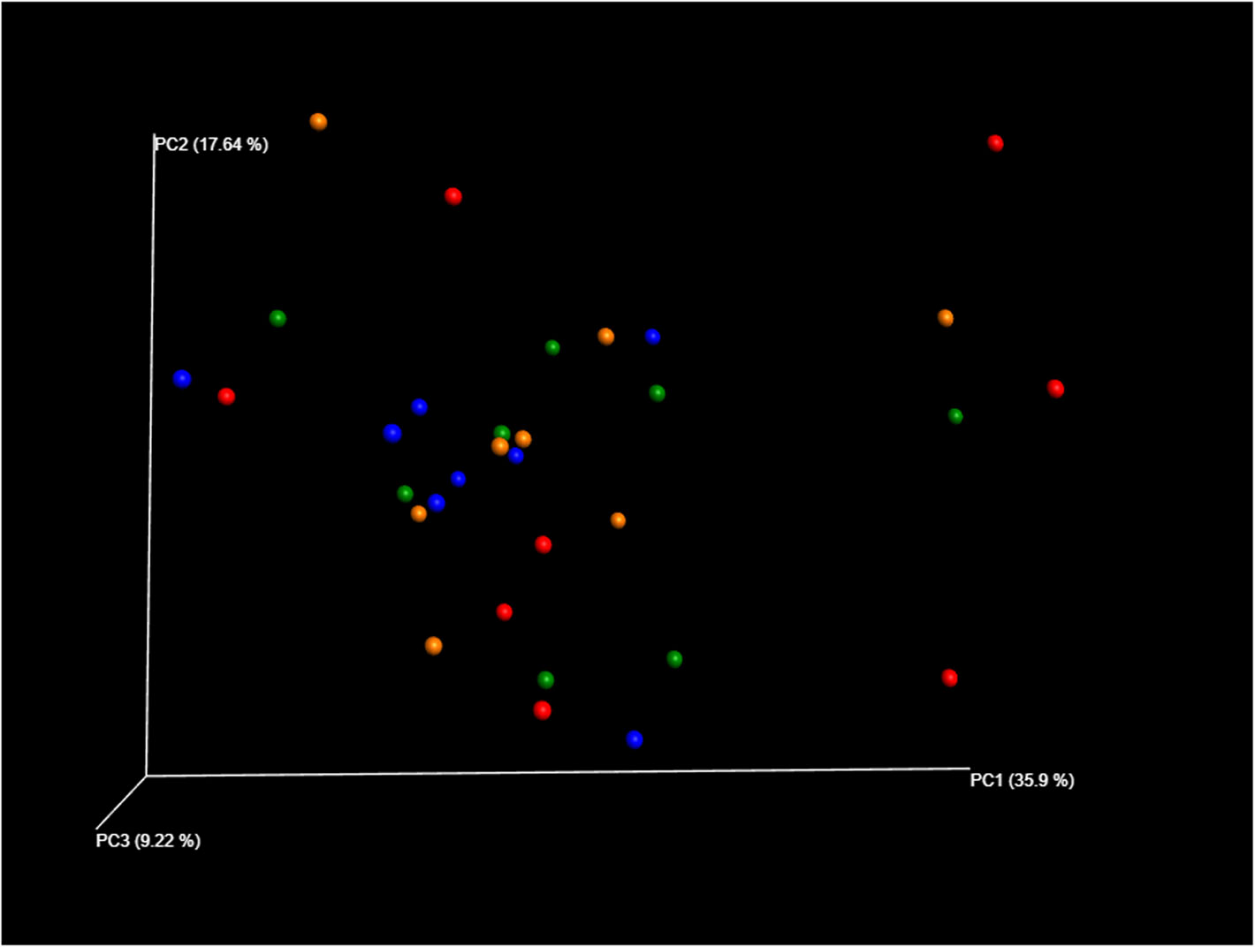
Principal component analysis of the microbial composition of the colonic contents. Every dot represents one of the 32 piglets analysed: 8 DS CON piglets (red), 8 DS HF piglets (blue), 8 RS CON piglets (orange) and 8 RS HF piglets (green)

**Fig. 2:**
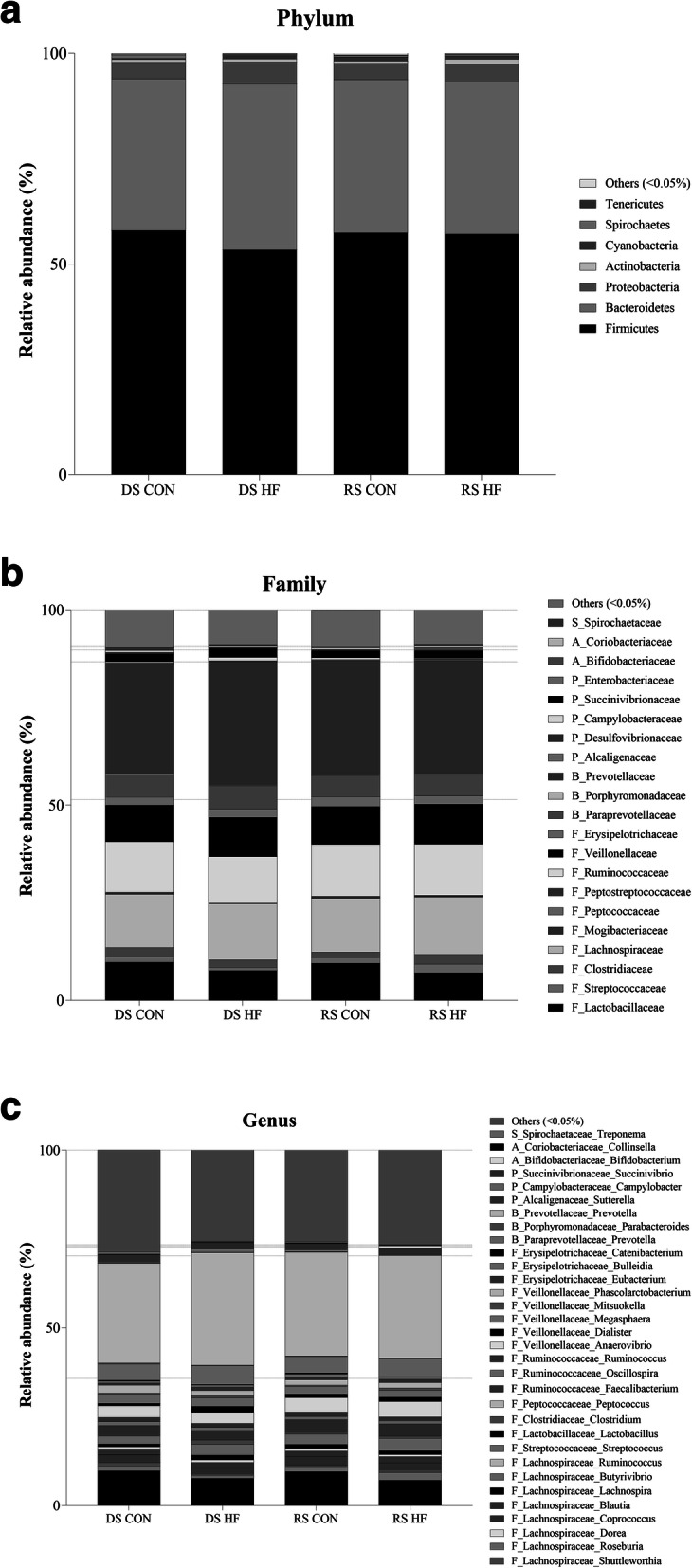
Proportion of the main abundant phyla (**a**), families (**b**) and genera (**c**) in the different pig groups (*n* = 8/treatment). Only phyla, families and genera with a relative abundance higher than 0.05% are displayed. Dashed lines in Fig. 2**b** and Fig. 2**c** separate phyla. F=Firmicutes, B=Bacteriodetes, P=Proteobacteria, A = Actinobacteria, S=Spirochaetes

No significant differences in microbiota diversity or richness were observed between maternal and piglet dietary treatments (*p* < 0.10). The global microbiota composition was not affected by the sow or piglet treatment, as observed by the PCoA in the Fig. 1. At the phylum level, this was translated by no impact of sow diet or HF challenge on the relative abundance of the different phyla with a relative abundance of more than 0.05%, as presented in Fig. 2a. The ratio between Firmicutes and Bacteroidetes was not impacted (*p* > 0.05) by the sow or piglet treatments (1.71 ± 0.18 for DS CON; 1.39 ± 0.09 for DS HF; 1.58 ± 0.14 for RS CON; 1.64 ± 0.14 for RS HF). Also on the family and genus level no significant differences were seen when looking at those families and genera with a relative abundance of more than 0.05% (Fig. 2b, Fig. 2c).

### Genes identified by DE analyses

After inspection of the datasets, for both the colon mucosal dataset as well as the liver dataset, one sample was dropped (Supplementary Figure F2A and F2B). For both datasets it belonged to a piglet that received a control diet and whose mother received an RS diet, although it was not the same piglet for both tissues. DE analyses were therefore performed between 16 DS and 15 RS piglets for the sow treatment comparison and between 15 CON and 16 HF piglets for the piglet treatment comparison. In the colon mucosal dataset, the greatest differences were found between the groups that received a different maternal diet (Additional file 4). A total of 12 genes were found to be significantly differentially expressed, with 4 genes more expressed in the RS group compared to the DS group, and 8 genes more expressed in the DS group versus the RS group. In the liver, with regard to the maternal diet, 10 genes were upregulated in the RS group compared to the DS groups and 12 genes were more expressed in the DS group. Between piglet treatments, the liver showed a more distinct DE of genes. One hundred ninety-two genes were overexpressed in the piglets that received a high fat diet compared to those that got the control diet. Two hundred twenty genes were more expressed in the CON group compared to the HF group. Nevertheless, the list of overrepresented genes in either the CON or HF group exposed not many meaningful GO terms. We confirmed two genes, *ASNS* and *ELOVL2*, that were two-fold upregulated in the HF group using qPCR. This resulted in an upregulation of 1.74 for ASNS (*p* < 0.001) and of 2.09 for ELOVL2 (*p* < 0.001). An overview of the functional enrichment in liver between the CON and HF group is shown in Additional file 5.

### Significant co-expressed gene modules identified using WGCNA

**Fig. 3:**
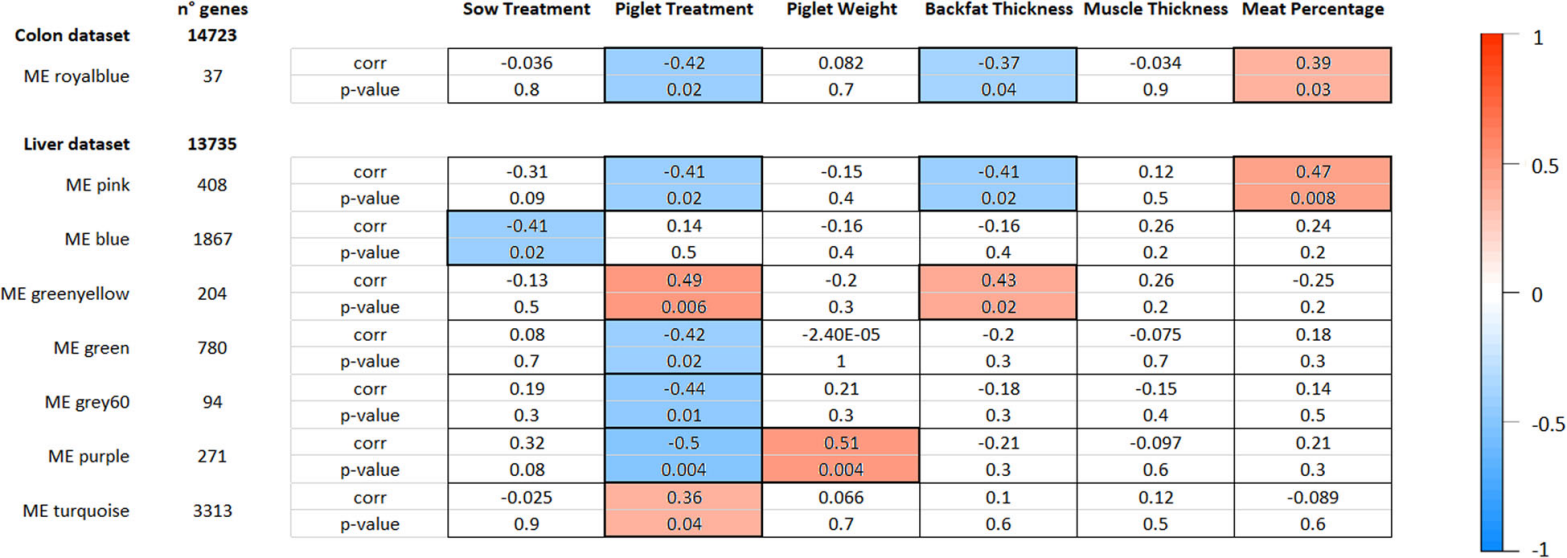
Overview of correlations of the significant modules in colon and liver. Correlations were calculated between the eigenvalue of the module and traits of interest: sow treatment (DS as 0, CON as 1), piglet treatment (CON as 0, HF as 1), piglet weight, backfat thickness, muscle thickness and meat percentage

To obtain scale-free topology networks that featured a suitable amount of node connectivity, the soft threshold chosen was set at 10 for the colon mucosal dataset and at 4 for the liver tissue dataset. In the colon mucosal dataset, there were 21 modules created. None of these modules were significantly correlated with sow treatment. However, one module (royalblue) was significantly correlated with piglet treatment (*p* = 0.02) as well as with back fat thickness (*p* = 0.04) and meat percentage (*p* = 0.03) (Fig. 3). For this module the genes showed an overall lower expression in the HF piglets compared to the CON piglets and their expression levels were negatively correlated with back fat thickness and positively correlated with meat percentage, which is in concordance with the direct effect the HF diet has on back fat thickness and meat percentage. In the liver dataset there were in total 22 modules of which 1 module was significant for sow treatment (blue, *p* = 0.02) and 6 were significant for piglet treatment (pink, *p* = 0.02; greenyellow, *p* = 0.006; green, *p* = 0.02, grey60, *p* = 0.01; purple, *p* = 0.004; turquoise, *p* = 0.04) (Fig. 3). For these 6 modules, back fat thickness and meat percentage showed an expected pattern with a correlation in the same direction for back fat thickness and in the opposite direction for meat percentage, albeit not always showing a significant correlation. Correlation values of additional modules are shown in Supplementary Figures 3A and 3B. An overview of all the functional gene ontology terms that are overrepresented or underrepresented in the significant modules (*p* < 0.05) for the colon mucosal or liver dataset is given in Additional file 6.

### Combining cholesterol and triglycerides analyses with WGCNA analyses

**Fig. 4:**
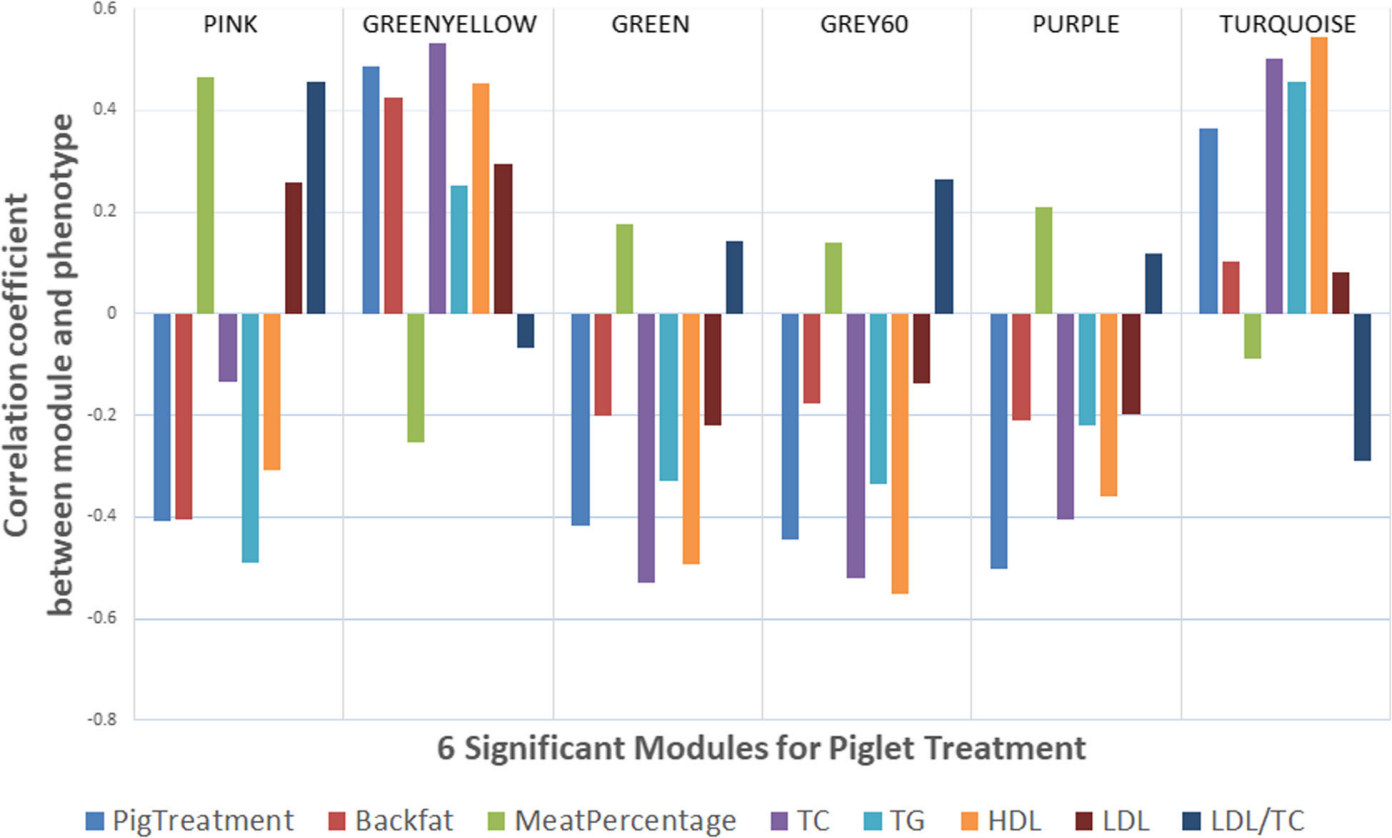
Correlations between the 6 significant WGCNA modules for piglet treatment in the liver dataset with piglet treatment (CON as 0, HF as 1) with back fat thickness and meat percentage, as well as total cholesterol (TC), triglycerides (TG), high density lipoprotein (HDL), low density lipoprotein (LDL) and ratio (LDL/TC) in pigs’ fasted serum after 6 weeks of HF challenge

Of the 31 animals of which liver samples were analysed using WGCNA, 26 had cholesterol values measured. For these 26 animals, the eigenvalues of all significant modules for piglet treatment were correlated with the cholesterol values TC, TG, HDL, LDL and LDL/TC. Figure 4 highlights the 6 modules in the liver dataset that were significant for piglet treatment with regard to their correlations with specific phenotypes for these 26 animals. This figure shows the correlations between these modules and the cholesterol values measured. In addition, the correlations between the modules and back fat thickness and meat percentage are shown since Table 1 revealed that HF diet was significantly different for these phenotypic measurements. The correlations with back fat thickness and meat percentage for these 26 animals were the same as for the 32 animals measured and reported in Fig. 3.

### Combining DE analyses with WGCNA analyses

**Fig. 5:**
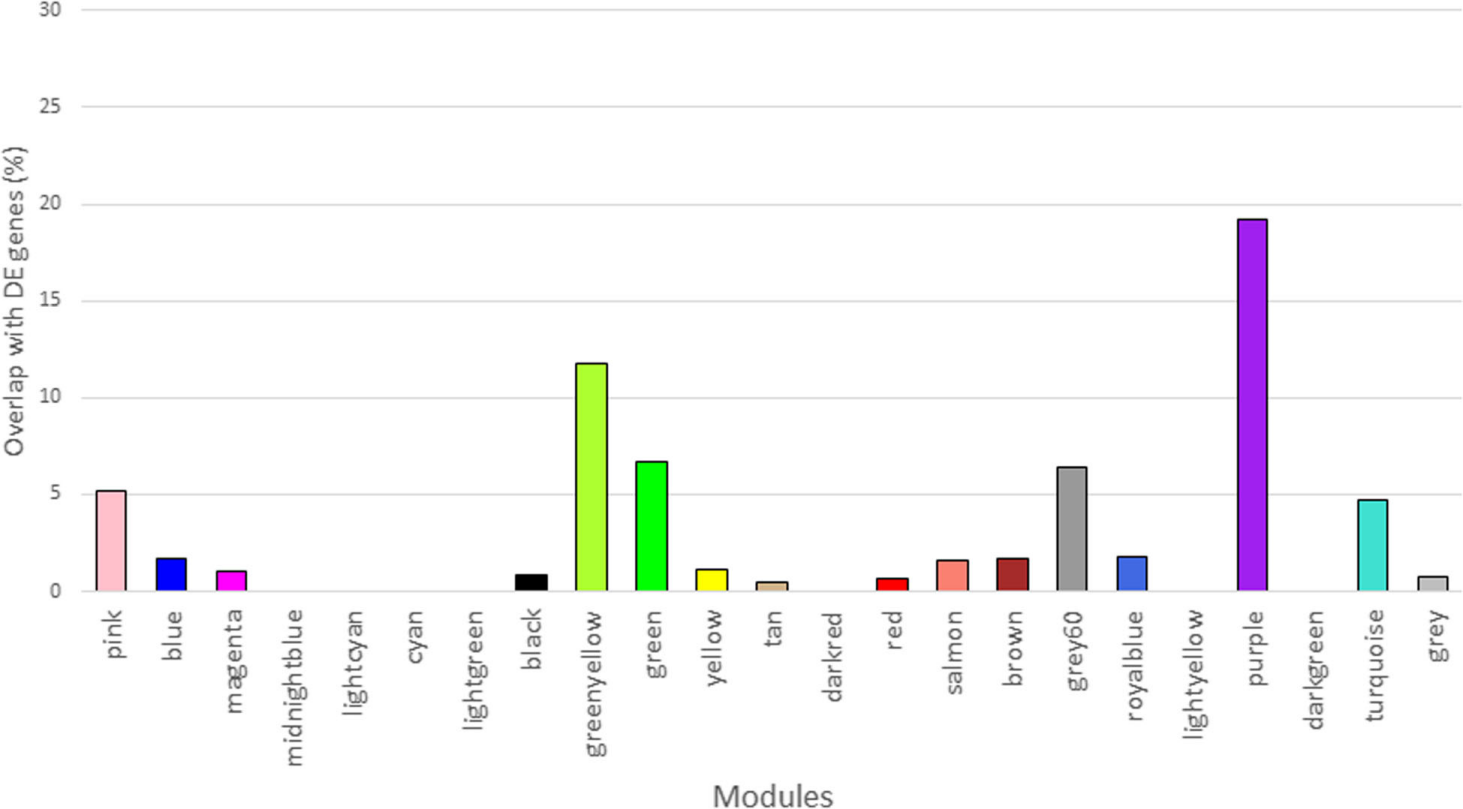
Overlap between modules and DE expressed genes for piglet treatment in liver dataset. The coloured bars represent the percentage of genes in a module that are differentially expressed in the liver between piglets that received a control diet (CON) and those that received the high fat diet (HF). Modules that were significantly correlated with piglet treatment were pink (*p* = 0.02), greenyellow (*p* = 0.006), green (*p* = 0.02), grey60 (*p* = 0.01), purple (*p* = 0.004) and turquoise (*p* = 0.04)

In the liver, a total of 412 genes were DE due to piglet treatment. When comparing this DE list with the list of different WGCNA modules in the liver, it could be noted that for the purple module, which was annotated for GO terms such as lipid metabolism, 52 genes of the in total 271, or 19%, were in common. When looking at all other modules in relation to the DE list for piglet treatment in the liver dataset, this module is in percentage terms the strongest present in the DE list, followed by the greenyellow (12%), green (7%), grey60 (6%), pink (5%) and turquoise (5%) module (Fig. 5). These modules were expectedly the ones that had the largest absolute correlation values with piglet treatment. All the remaining modules had between 0 and 2% of their genes present in the DE list.

## Discussion

Feeding RS is described to boost the immune system, mainly by influencing microbiota composition and increasing the production of beneficial SCFA such as butyrate due to these microbial changes [20]. Studies on the direct immunomodulatory effects of RS are rare, but it is also known that non digestible carbohydrates could bind to specific receptors on cells of the immune system and act in a microbiota-independent way on the immune system [21]. Due to the direct and indirect positive effects on the immune system, and as an attractive alternative to antibiotics to improve animal health, RS has been studied as prebiotic in pig feed [4, 22]. However, to our knowledge, not much work has been done on the maternal impact of RS on the metabolism and the microbiota of the progeny. Therefore, we designed an experiment given RS or DS to sows during late gestation and lactation and looked at the impact on the progeny.

First of all, piglet performance at the time of weaning, measured by body weight and intestinal morphology, did not expose any improvements [16]. We found that this absence of weight differences continued every week after weaning till 10 weeks post-weaning, even when piglets were provided a high fat diet. With regard to microbiota differences, Leblois et al. (2018) described microbial differences during gestation in the sows of our experiment, which translated in a higher Firmicutes to Bacteroidetes ratio, but these changes disappeared during lactation [16]. This is contradictory with Yan et al. [23], who also fed RS as a maternal diet, but noted a distinct microbial profile between their two groups of sows of low RS and high RS at the end of their lactation period. This could be due to the fact that the RS in our study has been given at another period during gestation. While Yan et al. [23] provided the RS diet from 30 days to 108 days of gestation, in this study, sows were fed the RS at the late gestation starting at day 88 and during the lactation period. Another difference is the source of RS. While in the study by Yan et al. [23] the RS came from amylose corn, our RS source was pea starch. We chose to give RS in late gestation and lactation since similar studies have shown potential maternal dietary effects given during this period [11, 24, 25]. When looking at the progeny, no major differences in microbial community were found, not at the moment of weaning [16], nor at 10 weeks post-weaning. This lack of major microbial differences in the progeny agrees with Arnal et al. [26] who fed antibiotics during late gestation and lactation and did not see any impact on the offspring’s colonic microbiota in the short or longer term.

Even though the microbiota was not affected by the maternal diet, the total SCFA production was higher in piglets born from RS sows. This is in line with Le Bourgot et al. [27] who fed short chain fructooligosaccharides to sows and did not immediately see differences between groups of piglets during the suckling phase, but reported higher SCFA production after weaning at day 90. Rather than looking at the microbial profile, it could therefore also be of interest to look at the metatranscriptome, to investigate possible differences in the function of the bacteria.

Gene expression analyses through RNA-seq with regard to maternal RS during late gestation and lactation resulted in only a relative small number of genes DE in both liver and colon, which did not allow us to perform any meaningful functional annotation or pathway analyses. One set of genes in the liver however was significantly negatively correlated with maternal treatment. This module counted 1867 genes (blue module, *p* = 0.02), but was not enriched for genes involved in immune function or lipid metabolism, even though these gene ontology terms are often reported when examining the direct dietary effect of RS to pigs [4, 5, 8, 28, 29].

It is not surprising that a more distinct transcriptomic difference between CON and HF piglets was found since this dietary treatment was a direct treatment given to the piglets themselves as opposed to the maternal diet. It can however not be excluded that transcriptomic differences or the differences found in parameters such as back fat thickness, meat percentage or cholesterol measurements between the high fat and control diet were not only due to the fat content of the diet, but were also a reflection of differences in feed intake, although this was not measured during the experiment. The most significant module of genes correlated with piglet treatment was found in the liver (purple module, *p* = 0.004) and was annotated with terms such as lipid metabolic process, fatty acid beta-oxidation, fatty acid metabolic process and fatty acid biosynthetic process (Additional file 6). Figure 5 shows that this module has also the most overlap with the list of DE genes. Fifty-two genes in this module were also significantly DE when comparing HF with CON diet and 33 of those were significantly down-regulated in the HF diet, while 19 were significantly up-regulated in the HF diet. The module’s eigenvalue was negatively correlated with piglet treatment which means that the overall expression of the 271 genes in this module was lower in those animals that received the HF diet, however, that does not mean that individual genes in this module were always lower expressed due to the HF diet. When looking at maternal diet, this module showed a trend (*p* = 0.08) towards significance for maternal treatment, with a positive correlation towards the RS diet. This would mean that this cluster of genes would be more explicit in those animals whose mother received the RS diet.

One of the genes that belonged to the intersection of DE genes and the genes in the purple module and that was upregulated in the HF piglets, was the gene translating in Asparagine Synthetase (*ASNS*); which was previously reported to be upregulated in the liver of mice fed a high-fat diet [30]. Another gene encoded for Fibroblast Growth Factor 21 (*FGF21*), and was more than 4-fold higher expressed in the HF animals. Subcutaneously injected FGF21 has been described to decrease food intake to 50% and therefore reduce body weight in minipigs [31]. FGF21 regulates triglyceride metabolism and it is shown that overexpression of *FGF21* finally would lead to a decline in lipid accumulation [32]. FGF21 is also found to increase insulin sensitivity and endogenous FGF21 is believed to protect obese individuals against insulin resistance [33, 34]. Another gene belonging to the purple module was the gene for Elongation of Very Long Chain Fatty Acids protein 2 (*ELOVL2*), which showed a two-fold upregulation due to the HF diet. In trout, *ELOVL2* was upregulated in the intestine in the genetic line selected for high fat content [35]. *ELOVL2* was also found to be a hub gene for a module of co-expressed genes that was significantly correlated to insulin secretion regulation [36]. It seems as if the overexpression of genes such as *FGF21* and *ELOVL2* in our experiment are a means for the animal to cope with the metabolic impact of a HF diet. Other genes, whose expression was downregulated in the HF diet group also interfered with insulin mediated glucose uptake. *FETUB*, for instance, is involved in the insulin uptake regulation [37]. Elevated levels of fetuin-B, translated by the *FETUB* gene, have been described as associated with insulin resistance [38]. Other genes that were significantly downregulated due to the HF diet were for example PKLR and FMO1. The pyruvate Kinase (*PKLR*) gene shows significant associations with Type 2 Diabetes [39]. Flavin containing monooxigenases activity and *FMO* mRNA levels were elevated in diabetic rats [40]. A possible explanation for these upregulated genes in our CON piglets compared to the HF piglets could lay in the fact that the CON diet contained 10% maize starch while in the HF diet this was substituted by 10% palm oil.

The second most significant module was also found in liver due to piglet treatment. The greenyellow module (*p* = 0.006) had immune system process and immune response as the most significant GO terms. The module counted 204 genes. Using Gorilla, it could be noted that this cluster was pointing more towards the adaptive immune system with a positive regulation of lymphocyte activation, with genes such as CD38, CD1D, MAP3K8, CCL5, LCK, CD40, HAVCR2, TNFSF13B, ICOS, BTK, IL7R, CD86, and CD80. The expression of these genes showed an overall higher level in the piglets receiving the HF diet. Pathway analysis showed an inflammation mediated response through chemokine and cytokine signalling as well as T cell activation. Indeed, T cells play an important role in liver diseases [41, 42]. However, this module was not significantly (*p* = 0.5) correlated with maternal diet, meaning that this cluster of genes would be showing the same expression pattern, regardless of maternal diet.

Another immune function annotated module was the royal blue module in the colon dataset, which was significantly correlated with piglet treatment and counted 37 genes. This module was enriched for the immune functions ‘response to interferon gamma’. The module shows an overall higher expression of these 37 genes in the control group compared to the HF group. Genes amongst those belonging to this module were for instance PSME1, PSMB8, PSMB9, IL15, NOS2, UBD, NLRC5, ARSA, GBP1, GBP2, IRF1, TAP1, TAP2, TAPBP, and GM2A. However, none of these genes was differentially expressed. Correlating the eigengene of this royal blue module with the expression values of IFNG results in a positive correlation coefficient of 0.59. IFNG itself was also not DE, and did not belong to a module. Again, no correlation was seen with maternal diet (*p* = 0.8).

In summary, only a handful of genes were DE in liver due to maternal diet and only one set of genes in the liver was significantly negatively correlated with the maternal treatment (blue module). Both these lists of genes did not reveal any clear functional annotation. Therefore, it seems that, under the tested conditions, no clear metabolic programming in the liver of the progeny has occurred due to maternal RS treatment. Likewise, when looking at differences on the intestinal level, the intestine did definitely not seem to be prone to a maternal programming, as not many genes were DE, and no modules were correlated with maternal diet. This lack of intestinal programming is in contrast to other studies investigating the influence of maternal diet additives on gut health in progeny [11–13]. Possibly source, dose, timing and duration of the given RS prebiotic could influence the effectiveness of this programming on a metabolic and/or intestinal level and these are therefore important factors to take into account when trying to induce maternal programming.

## Conclusions

In conclusion, no clear effect of maternal dietary RS with regard to gut health could be translated to the progeny when given during late gestation and lactation. Minor changes were seen in the SCFA production, but the gut microbiota did not change majorly and there were not many genes differentially expressed due to the maternal diet, not in colon, nor in liver. A high fat diet given to the progeny after weaning does however evoke metabolic changes in the liver, but RS was not able to significantly influence that, not for the better nor for the worse.

## Methods

### Animals, diets and housing

Twenty-four Landrace sows from the Walloon Agricultural Research Centre (CRA-W) in Gembloux (Belgium) were used during this animal experiment; they were artificially inseminated with Piétrain semen coming from 3 sires, and they were housed in group on straw from 3 days after until 1 week before farrowing. From then on, they were housed in individual farrowing units equipped with wood shavings and a heat lamp for piglets. Sows and piglets up till the moment of weaning were housed at the CRA-W. All animal procedures were approved by the ethical committee of the University of Liège (protocol n°1661). Sows were fed a standard diet during gestation until day 88. At day 88 of gestation, sows were divided in two groups, one group (*n* = 12) received a diet containing 33% of standard maize starch, considered as digestible (DS) while the other group (*n* = 12) received a diet rich in pea starch (Nastar, Cosucra, Belgium), considered as resistant starch (RS) until the end of lactation (28 days after piglet birth). The diets were adapted for nutritional requirements of sows for the gestation and lactation period [16]. The division in groups was made taking the semen used for mating and the sow’s parity into account. Within the DS diet, 4 sows were of the first parity (P1), 2 sows were of second parity (P2), 3 sows were of third parity (P3) and 3 sows had a higher parity or equal to 4 (*P* ≥ 4). Within the RS group, the parities distribution was similar: 4 P1 sows, 2 P2 sows, 4 P3 sows and 2 *P* ≥ 4 sows. Sows were fed according to their parity to follow a specific pattern in body weight increase. On average, the number of piglets born alive was 13.1 ± 0.9 (mean ± SEM) for RS fed sows and 13.2 ± 0.7 for DS fed sows. Piglets stayed with their respective mother until weaning at the age of 28 days. At weaning, 44 female piglets were moved to the Centre for Animal Production at the University of Liège in Gembloux. To get to 22 piglets per maternal diet, an even number of piglets (two or four) was selected of 6 sows per maternal treatment. Piglets were selected based on weights closest to the average of the litter at time of weaning. The temperature on the day of arrival was 26 °C, piglets were housed on grates, two littermates being housed in each pen. Piglets were fed a standard weaning diet during 21 days, devoid of antibiotics, organic acids or non-starch polysaccharide enzymes. At day 22 post-weaning, littermate piglets were divided into two groups: one group was fed a control diet (CON) containing 10% of maize starch (Roquette, Lestrem, France), while the other group received a high fat diet (HF) containing 10% of palm oil (Mosselman S.A., Ghlin, Belgium) to induce a high fat challenge. During this challenge, the feed provided was a grower 1 diet (11-25 kg) followed by a grower 2 diet (25-50 kg) to reach the nutritional requirements considering bodyweight (Table 4). Both diets were fed ad libitum till the end of the experiment.

**Table 4:** Ingredients and analysed chemical composition of grower diets fed to piglets

Ingredient (%)	Grower 1 (11–25 kg)		Grower 2 (25–50 kg)	
	CON	HF	CON	HF
Barley	17.7	17.7	11.25	11.25
Maize	13.5	13.5	9.4	9.4
Wheat	13	13	27	27
Soybean meal 48%	12.1	12.1	14.2	14.2
Palm oil	–	10	–	10
Maize starch	10	–	10	–
Golden soy	9	9	0	0
Nutex 68 (Dumoulin Inc)	4.5	4.5	0	0
Bread flour	4.5	4.5	4.95	4.95
Biscuit flour	4.5	4.5	4.95	4.95
wheat bran	3.6	3.6	2.34	2.34
Rapeseed cake	2.2	2.2	4.95	4.95
Sunflower cake	0	0	3.15	3.15
Chalk	1.5	1.5	1.36	1.36
Beet pulp	1.3	1.3	1.8	1.8
Fat	0	0	1.67	1.67
Maize gluten	0	0	1.188	1.188
Amino acids (Thr, Try, Met, Lys)	1.132	1.132	0.775	0.775
Minerals & Vitamins	0.603	0.603	0.369	0.369
Salt	0.4	0.4	0.333	0.333
Molasses	0	0	0.36	0.36
**Analysed chemical composition (%)**				
Dry Matter	88.8	89.7	89.94	89.41
Crude Protein	18.03	17.81	18.40	18.84
Ether Extract	5.05	15.94	4.77	14.88
Acid Detergent Fibre	5.15	5.55	5.29	5.37
Neutral Detergent Fibre	12.69	13.83	13.65	14.65

The animal experiment ended 10 weeks post-weaning. Five piglets (2 from the DS CON group, 2 from the DS HF group and 1 from the RS CON group) facing too severe weight loss after weaning had to be treated with antibiotics and were thus taken out of the experiment, while one pig (from the RS CON group) died by sudden death.

### Growth performance measurements and sampling

For all the remaining 38 piglets, bodyweight was recorded weekly during the whole experiment. Six weeks after the beginning of the high fat challenge, piglets were fasted for 12 h and blood was sampled in 9-ml serum tubes (S-monovette, Sarstedt, Germany). After centrifugation at 2000 g for 10 min, serum was collected and stored at −20 °C until cholesterol components were measured. Two days before slaughtering, back fat, muscle thickness and meat percentage were determined. Back fat and muscle thickness were measured at the last rib at 60 mm from the midline. Back fat was measured using the Renco LEAN-MEATER® (S.E.C. Repro. Inc., Quebec, Canada) and muscle thickness was measured by the Vetko plus (Noveko, Quebec, Canada). At the end of the experiment (10 weeks post-weaning), the piglets were anesthetized with a mixture of xylazine and zoletil 100 (4 mg of xylazine, 2 mg of zolazepam and 2 mg of tilamine/kg) and euthanized by an overdose of isoflurane followed by exsanguination. Content of the caecum, proximal colon (first part of the large intestine after the ileocaecal valve) and distal colon (last part of the large intestine before the rectum) was collected in sterile tubes and stored at − 80 °C until further analyses of the SCFA and microbiota. Mucosa of the proximal colon was obtained after rinsing the colon with a saline solution and scraping it on a cold surface. The mucosa was then snap-frozen in liquid nitrogen and stored at − 80 °C until RNA extraction. Also liver tissue was snap-frozen and stored at − 80 °C until RNA extraction.

### Cholesterol and triglycerides measurements

For 28 piglets (7 per sow/piglet dietary treatment combination), serum total cholesterol (TC), triglycerides (TG) and high density lipoprotein (HDL) were analysed with TRIGL, CHOL2 and HDLC3 packs on a Cobas 8000 instrument (Cobas, Roche, Switzerland). The low density lipoprotein (LDL) concentration was calculated following the formula by Friedewald et al. [43]: LDL = TC-HDL-TG/5.

### Short-chain fatty acid (SCFA) and microbiota composition

Of all 38 piglets that made it to the end of the trial short-chain fatty acids of piglets’ caecal and colonic contents were determined using HPLC as described earlier [16]. DNA was extracted from the proximal colon of 32 piglets, 8 per sow/piglet treatment combination and 16S rRNA sequencing was performed as described by Leblois et al. [16]. In short, the QiagenQIAamp stool Minikit (Qiagen, Germany) was used following the manufacturer’s protocol, preceded with two additional bead beating steps to improve sample homogenization. Sequencing was performed by DNA-Vision (Belgium) using the Illumina MiSeq (2 × 300 nt) and amplifying the V3-V4 region (forward primer: 5′-TCGTCGGCAGCGTCAGATGTGTATAAGAGACAGCCTACGGGNGGCWGCAG-3′ and reverse primer: 5′-GTCTCGTGGGCTCGGAGATGTGTATAAGAGACAGGACTACHVGGGTATCTAATCC-3′). Bioinformatics on the raw sequences was performed as previously described by Leblois et al. [16]. Also in this set of samples, variable numbers of sequences were found and therefore samples were rarefied to a similar sequence depth of 47,788 reads per sample.

### Statistical analyses

For growth performance parameters, cholesterol component levels and the SCFA quantities in the caecal and colonic contents statistical analyses were performed with SAS 9.2 software (SAS Inc., USA) using the MIXED procedure of SAS, including the maternal diet and the piglet treatment in a two-way ANOVA. Normality of the data was tested using the Shapiro-Wilk’s test at a significance level of *p* = 0.05 and variance equality was assessed using the Levene’s test. Since normality of the data was not achieved for microbiota composition, the non-parametric NPA1WAY test of SAS was used, either considering the maternal diet, the piglet diet or both in a 4 treatment setup. Data was corrected for multiple testing using a Benjamini-Hochberg false discovery rate.

### RNA extraction, library construction and RNA-seq processing

Total RNA was extracted from proximal colon mucosal tissue and liver tissue using the ReliaPrep™ RNA Tissue Miniprep System kit (Promega, USA) according to the manufacturer’s protocol and adding a bead beating step. The RNA concentration was quantified using a NanoDrop ND-2000 spectrophotometer (Nano-Drop Technologies, USA) and the quality was assessed using an Agilent Bioanalyzer 2100 (Agilent Technologies, Inc., USA). RIN values were checked to be at least 8.0 before further processing. Using the Illumina TruSeq kit, libraries were constructed of the colon mucosal and liver tissue samples of 32 piglets in total, 8 per sow/piglet treatment combination. RNA-sequencing was performed at GIGA, at the University of Liège. Seventy-five bp single-end sequencing was performed using the Illumina HiSeq™ 2000 platform. Reads were aligned to the latest porcine reference genome available on NCBI: *Sus scrofa* annotation release 106. This annotation is based on the *Sus scrofa* genome assembly v11.1. Mapping was done using STAR [44]. Quality control was performed using FASTQC [45] and Picard Tools (https://broadinstitute.github.io/picard/).

### Differential expression (DE) analyses

After inspection of the expression data by heatmap analyses one sample was dropped for further analyses in both the colon mucosal and liver tissue dataset (Supplementary Figure 2A and 2B). Low reads were subsequently filtered out so that the remaining datasets consisted of only those genes that had at least 10 counts in 90% of the samples. For the liver this resulted in 13,735 genes, for the colon 14,723 genes passed the threshold. To find differentially expressed genes, the R package DESeq2 [46] was conducted on each tissue separately. A likelihood ratio test was used to test the interaction effect of sow treatment (maternal diet of DS versus RS) and piglet treatment (piglet diet of CON vs HF). Since no gene displayed a significant interaction effect, the final model used for DE analysis included only the main effects of sow and piglet treatment. Genes were considered as DE when the difference between treatments was significant after adjusting for multiple testing using Benjamini-Hochberg (FDR < 0.1) (Additional file 4).

### Validation through qPCR

RNA was reverse transcribed using GoScript TM Reverse Transcription Mix (Promega, USA), following the manufacturer’s instructions. Intron-spanning primers were found in literature or designed using Primer3 [47]. Primers are shown in Table 5.

**Table 5:** Primers used for qPCR validation

Gene	Primer Sequence (5′ -- > 3′)	Reference
ELOVL2	F: ATCTGGTGGTGTGTGCTGAA	Own design
	R: GGAAGATGAGACAGCCGAAG	
FGF21	F: GAAGCCAGGGGTCATTCAAA	Rosenbaum et al. [48]
	R: GGTAAACGTTGTAGCCATCC	
PPIA	F: ACTGCCAAGACTGAGTGGTAAG	Own design
	R: CAGAGCAGGAACCAACAGCG	
GAPDH	F: CATCCATGACAACTTCGGCA	Chatelais et al. [49]
	R: GCATGGACTGTGGTCATGAGTC	

qPCR was performed on a StepOne Plus (Applied Biosystems, USA) using SYBR Premix Ex Taq Tli RNAse H Plus (TakaraBio, Japan). The program used was a standard program with denaturation at 95 °C for 5 s, followed by 40 cycles of annealing at 60 °C for 30s and elongation at 72 °C for 30s. Primer efficiencies between 100 and 110% were obtained and primer specificity was checked through melting curve analyses.

### WGCNA analyses

For both datasets, weighted gene co-expression network analysis (WGCNA) was used to cluster highly correlated genes and to find modules whose expression was significantly correlated with the treatments [50]. Sow treatment correlations were made coding the control diet DS as 0 and the experimental diet RS as 1. Piglet treatment correlations were made coding the control diet CON as 0 and the experimental diet HF as 1. Modules were considered to be interesting when the nominal p-value for their correlation with the treatment was *p* < 0.05. Additionally, other traits were examined such as piglet weight, back fat, muscle thickness and meat percentage at 10 weeks of age to see if these traits were correlated with the significant modules for the main effects. A supplementary correlation analyses was performed between the eigenvalue of the modules for those samples of which also cholesterol parameters were measured in order to obtain knowledge on which modules were correlated with those parameters.

### Identification of gene functions and pathway analyses

Gene function and pathway analyses were performed using the gene ontology (GO) tool Panther (version 13.1) [51] with the built-in *Sus scrofa* reference list and the Panther pathway and Panther GO-slim biological process annotation datasets. Additionally, Gorilla GO [52] was used, for which we set *Homo sapiens* as the investigated organism and chose a two unranked lists of genes approach with as background list all the genes that had at least 10 reads in 90% of the samples. GO enrichment was done on all sets of DE expressed genes, separated by up- and downregulated genes, as well as on the modules of co-expressed genes that were significant for sow and piglet treatment. The cut-off for considering a function to be over-represented was set at an FDR < 0.05.

## Supplementary information

**Additional file 1: Supplementary Figure 1.** Piglet bodyweight from the moment of weaning at 4 weeks of age until 10 weeks of age. Piglet HF or CON diets were given starting at week 7. Digestible Starch (DS), Resistant Starch (RS), Control (CON), High Fat (HF).

**Additional file 2: Supplementary Figure2A and 2B.** Heatmap of all colon samples (A) and all liver samples (B) in our experiment. Sample C27 was dropped for further analyses in the colon dataset, and sample F23 was dropped for further analyses in the liver dataset. Both these samples belonged to the group of piglets whose mother received the RS diet and themselves received the CON diet.

**Additional file 3: Supplementary Figure 3A and 3B.** Overview of correlations of the all modules in colon (A) or liver (B). Correlations were calculated between the eigenvalue of the module and traits of interest: sow treatment (DS as 0, CON as 1), piglet treatment (CON as 0, HF as 1), piglet weight, backfat thickness, muscle thickness and meat percentage.

**Additional file 4 **Differential Expressed (DE) Gene Ontology (GO) term enrichment. Genes were considered as DE when the difference between treatments was significant after adjusting for multiple testing using Benjamini-Hochberg (FDR < 0.1). DE was investigated with regard to maternal diet (Digestible Starch (DS) vs Resistant Starch (RS) diet) and piglet treatment (Control (CON) vs High Fat (HF) diet). For all genes, the ‘log2 fold change’ and recalculated fold change is shown, as well as the ‘(adjusted) *p*-value’ for that fold change. Other metrics shown are ‘baseMean’, or the mean of the normalized counts for all samples, ‘lfcSE’, or the standard error estimate for the log2 fold change estimate and ‘stat’, the Wald statistic for the comparison examined.

**Additional file 5.** Differential Expressed (DE) Gene Ontology (GO) term enrichment. GO enrichment was performed on all differential expressed (DE) genes (FDR < 0.1) in the liver between piglets that received the control (CON) diet versus piglet that received the high fat (HF) diet. The GO enrichment tool Panther (version 13.1) was used and Pathways and Slim Biological Processes were examined.

**Additional file 6.** WGCNA Modules Gene Ontology (GO) term enrichment. GO enrichment performed on all differential expressed (DE) genes (FDR < 0.1) in the significant WGCNA modules of the colon or liver datasets. The GO enrichment tool Panther (version 13.1) was used and Pathways and Slim Biological Processes were examined.

## Data Availability

All relevant data generated or analysed during this study are included in this article and its additional files. All raw microbiota and RNAseq sequences were submitted to the European Nucleotide Archive database under the accession number PRJEB33554. The reference dataset used to annotate the RNA-seq data was the latest porcine reference genome available on NCBI: *Sus scrofa* annotation release 106, based on the genome assembly v11.1 (https://www.ncbi.nlm.nih.gov/genome/annotation_euk/Sus_scrofa/106/).

## Abbreviations

RS: Resistant Starch
SCFA: Short chain fatty acids
DS: Digestible Starch
CON: Control
HF: High fat
TC: Total cholesterol
TG: Triglycerides
HDL: High density lipoprotein
LDL: Low density lipoprotein
DE: Differential expression
WGCNA: Weighted gene co-expression network analysis
GO: Gene ontology
ASNS: Asparagine Synthetase
FGF21: Fibroblast Growth Factor 21
ELOVL2: Elongation of Very Long Chain Fatty Acids protein 2
FETUB: Fetuin-B
PKLR: Pyruvate Kinase
FMO: Flavin containing monooxigenases activity
