# Supplementary figures and images for: Maternal dietary resistant starch does not improve piglet’s gut and liver metabolism when challenged with a high fat diet
Source: BMC Genomics. 2020 Jun 26;21:439. doi: 10.1186/s12864-020-06854-x (PMC7318506; doi:10.1186/s12864-020-06854-x)

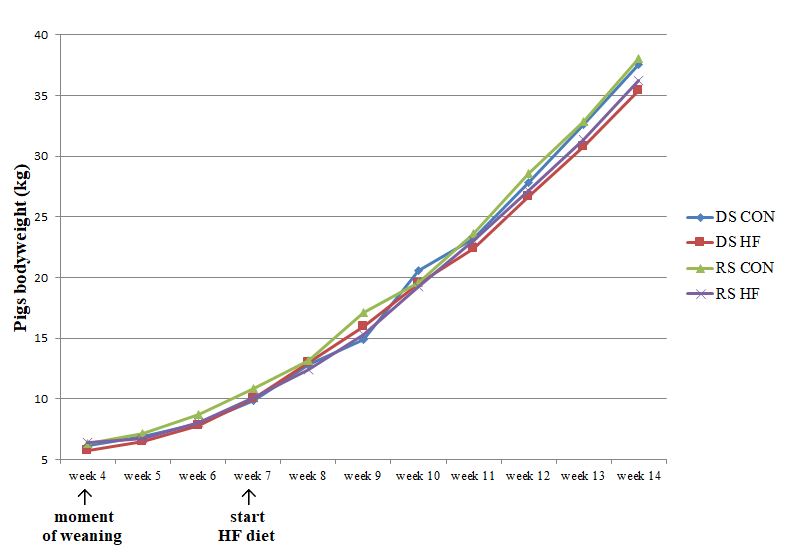

Supplement: Supplementary file 1 — Additional file 1: Supplementary Figure 1. Piglet bodyweight from the moment of weaning at 4 weeks of age until 10 weeks of age. Piglet HF or CON diets were given starting at week 7. Digestible Starch (DS), Resistant Starch (RS), Control (CON), High Fat (HF). [file 12864_2020_6854_MOESM1_ESM.png]

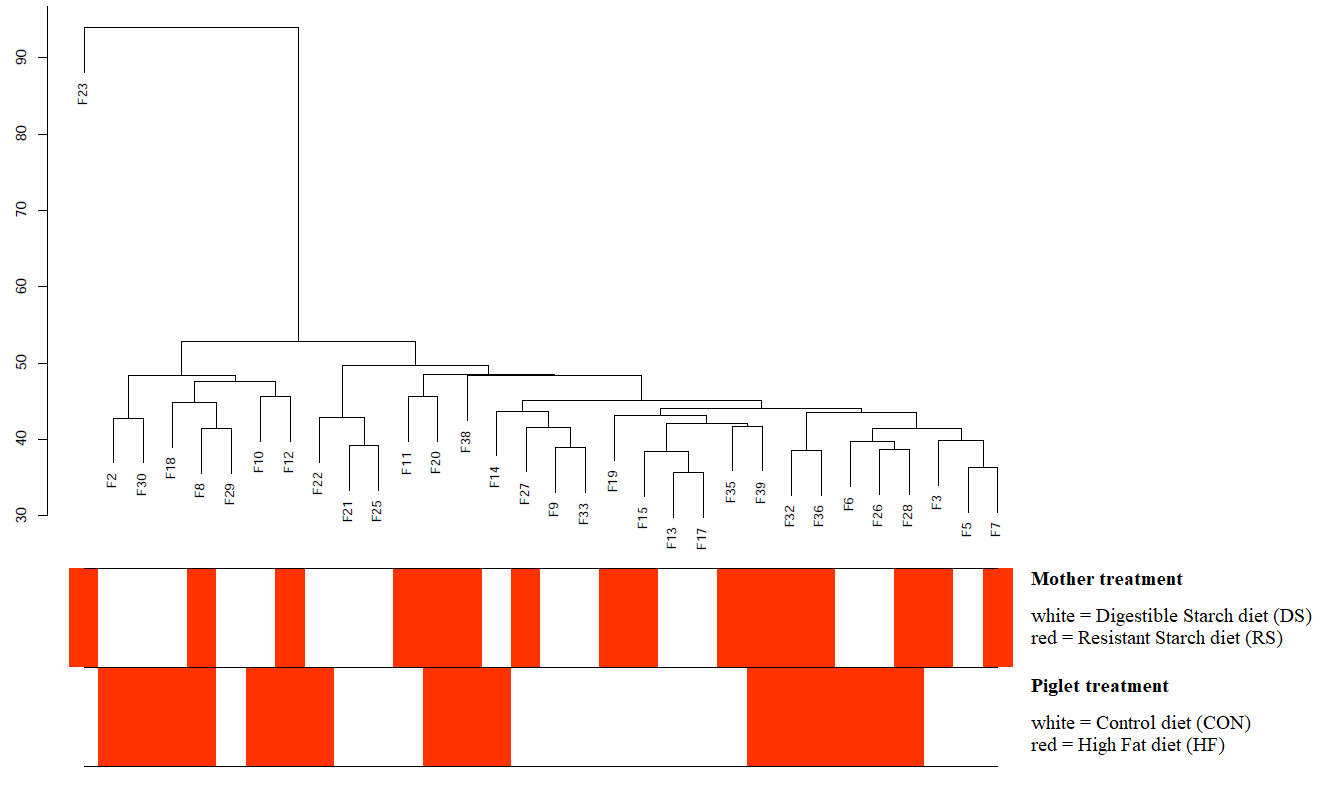

Supplement: Supplementary file 2 — Additional file 2: Supplementary Figure2A and 2B. Heatmap of all colon samples (A) and all liver samples (B) in our experiment. Sample C27 was dropped for further analyses in the colon dataset, and sample F23 was dropped for further analyses in the liver dataset. Both these samples belonged to the group of piglets whose mother received the RS diet and themselves received the CON diet. [file 12864_2020_6854_MOESM2_ESM.zip › Supplemental Figure F2B.png]

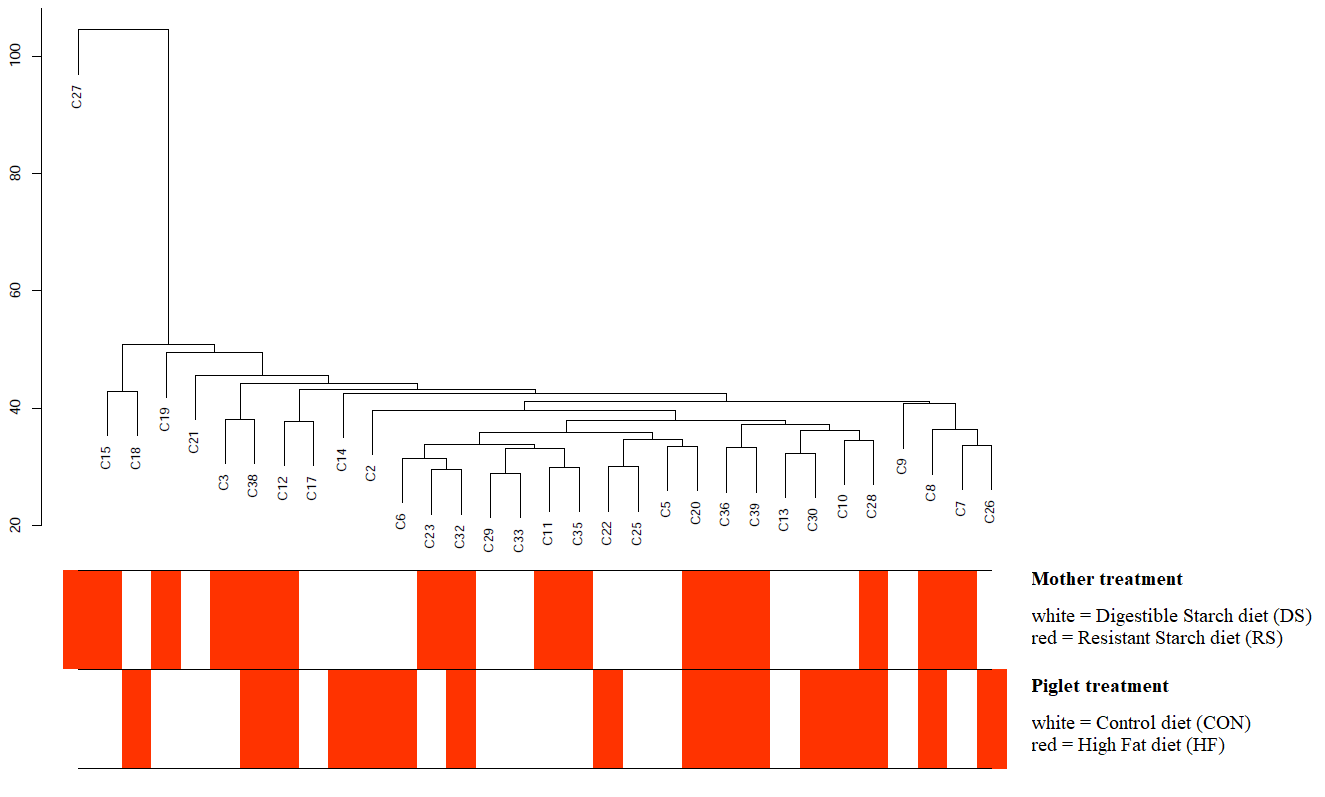

Supplement: Supplementary file 2 — Additional file 2: Supplementary Figure2A and 2B. Heatmap of all colon samples (A) and all liver samples (B) in our experiment. Sample C27 was dropped for further analyses in the colon dataset, and sample F23 was dropped for further analyses in the liver dataset. Both these samples belonged to the group of piglets whose mother received the RS diet and themselves received the CON diet. [file 12864_2020_6854_MOESM2_ESM.zip › Supplemental Figure F2A.png]

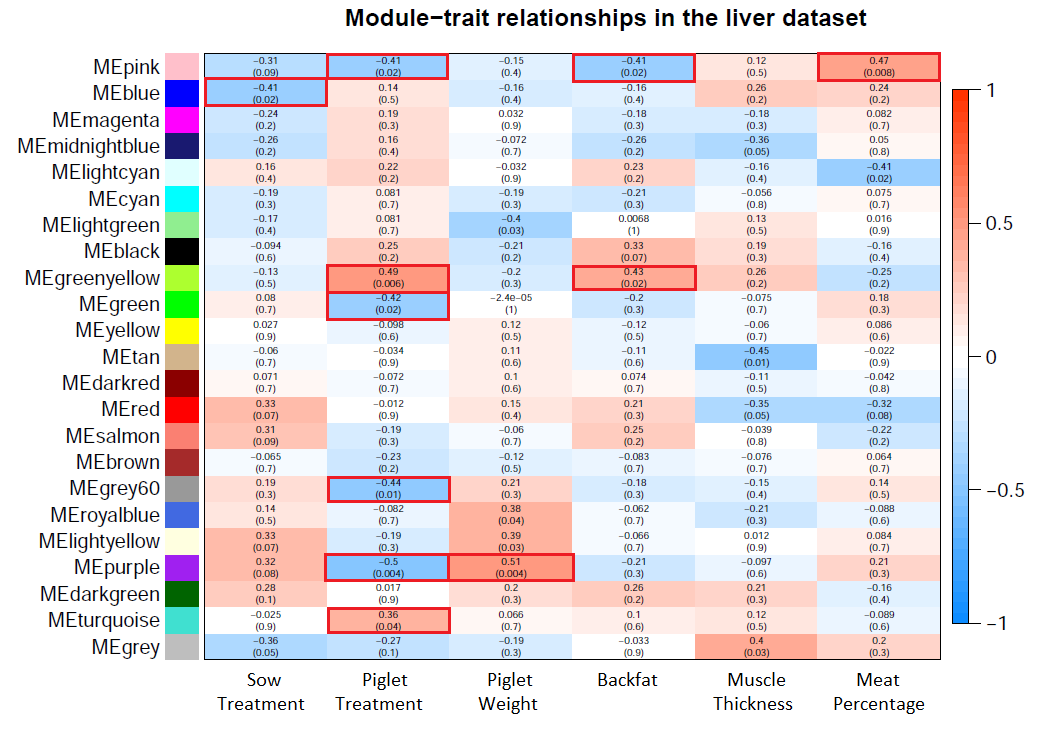

Supplement: Supplementary file 3 — Additional file 3: Supplementary Figure 3A and 3B. Overview of correlations of the all modules in colon (A) or liver (B). Correlations were calculated between the eigenvalue of the module and traits of interest: sow treatment (DS as 0, CON as 1), piglet treatment (CON as 0, HF as 1), piglet weight, backfat thickness, muscle thickness and meat percentage. [file 12864_2020_6854_MOESM3_ESM.zip › Supplemental Figure F3B.png]

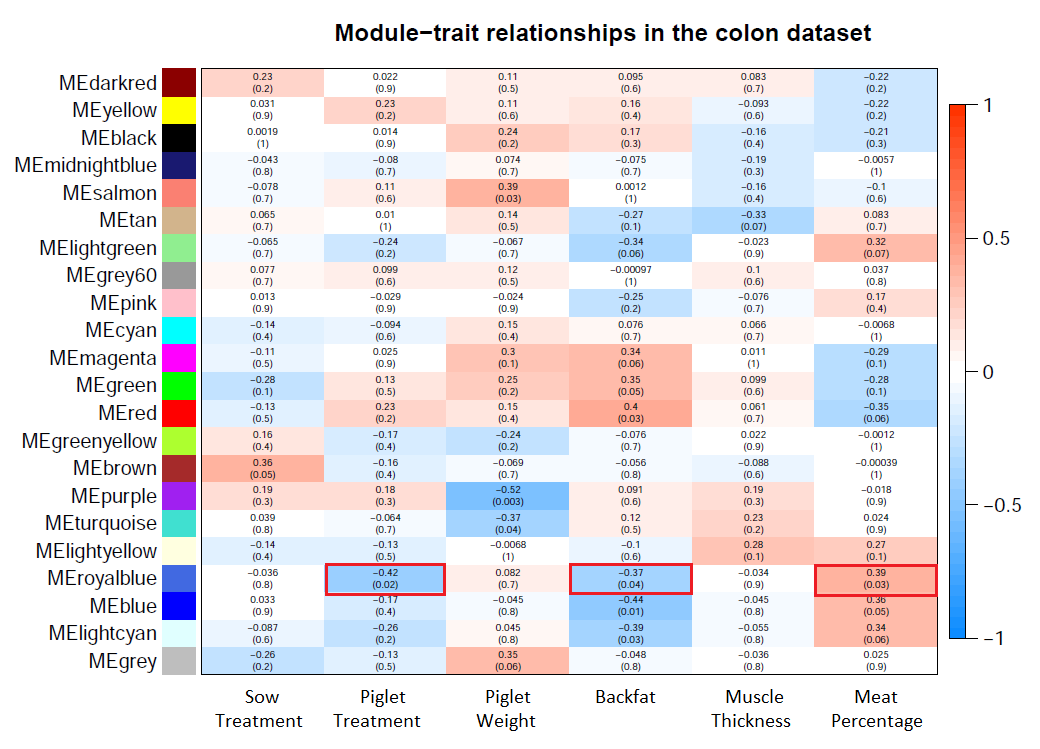

Supplement: Supplementary file 3 — Additional file 3: Supplementary Figure 3A and 3B. Overview of correlations of the all modules in colon (A) or liver (B). Correlations were calculated between the eigenvalue of the module and traits of interest: sow treatment (DS as 0, CON as 1), piglet treatment (CON as 0, HF as 1), piglet weight, backfat thickness, muscle thickness and meat percentage. [file 12864_2020_6854_MOESM3_ESM.zip › Supplemental Figure F3A.png]
